# HPV16E6-Dependent c-Fos Expression Contributes to AP-1 Complex Formation in SiHa Cells

**DOI:** 10.1155/2011/263216

**Published:** 2011-07-31

**Authors:** Feixin Liang, Shinichiro Kina, Hiroyuki Takemoto, Akira Matayoshi, Thongsavanh Phonaphonh, Nao Sunagawa, Keiichi Arakaki, Akira Arasaki, Hai Kuang, Hajime Sunakawa

**Affiliations:** ^1^Department of Clinical Neuroscience Oral and Maxillofacial Functional Rehabilitation, University of Ryukyus, 207 Uehara, Nishihara, Okinawa 903-0215, Japan; ^2^Department of Oral and Maxillofacial Surgery, Guangxi Medical University, Nanning 530021, Guangxi, China

## Abstract

To date, the major role of HPV16E6 in cancer has been considered to be its ability to inhibit the p53 tumor-suppressor protein, thereby thwarting p53-mediated cytotoxic responses to cellular stress signals. Here, we show that HPV16E6-dependent c-fos oncogenic protein expression contributes to AP-1 complex formation under oxidative stress in SiHa cells (HPV16-positive squamous cell carcinoma of the cervix). In addition, we examined the role of HPV16E6 in TGF-**α**-induced c-fos expression and found that the c-fos protein expression induced by TGF-**α** is HPV16E6 dependent. Thus, our results provide the first evidence that HPV16E6 contributes to AP-1 complex formation after both ligand-dependent and independent EGFR activation, suggesting a new therapeutic approach to the treatment of HPV-associated tumors.

## 1. Introduction

HPV (human papillomavirus) infection is a hallmark of uterine cervical cancer and is thought to be important, if not causal, for some types of tumorigenesis [1–3]. Two HPV-encoded proteins, E6 and E7, inactivate and inhibit the expression of the p53 and RB tumor suppressor proteins, respectively. Nevertheless, HPV infection is not sufficient for tumorigenesis, because HPV infection is also present in healthy individuals [4], and the overexpression of E6/E7 alone is capable of immortalizing primary human epithelial cells but does not induce tumor cell transformation [5]. Thus, understanding the conditions that stimulate HPV-infected cells is crucial for the development of effective treatments for HPV-associated tumors. 

The transcription factor AP-1, which is composed of heterodimers of members of the c-Jun and c-Fos families, regulates various cellular processes such as enhanced proliferation, apoptosis, and tumor metastasis [6]. In particular, among the various members of the AP-1 family, c-fos acts as a tumor promoter, and c-fos upregulation causes cellular transformation that is characterized by colony formation in soft-agar and tumor formation in nude mice [7, 8]. In addition, when c-fos binds to c-jun, it increases the gene expression of cyclinD1 and contributes to the potentiation of malignancy [9–11]. 

In cervical cancer, the AP-1 complex formed during tumor development consists of a c-fos/c-jun heterodimer [9, 12–14], and c-fos/c-jun formation is also implicated in HPV-induced esophageal tumor development [15]. In fact, various c-fos target genes are reported to be expressed at higher levels in cervical cancer cells with comparison to normal cervical epithelial cells [16]. While c-fos showed very low expression in normal samples and moderate expression in cervical premalignant lesions, the tumor tissues showed very strong expression [13]. It is, therefore, reasonable to assume that c-fos regulation plays a fundamental role in HPV-induced tumor development. However, the extracellular conditions in which c-fos expression and AP-1 complex formation are induced in HPV-infected cells are unknown. 

A shift in the composition of AP-1 from fra-1/c-jun to c-fos/c-jun heterodimers occurs during HPV-infected tumor development [7, 14]. The same shift in AP-1 composition also takes place under oxidative stress, such as after UV-B exposure [10]. As transient transfection experiments showed that HPV16E6 induces the transcription of the c-fos promoter [17], we hypothesized that under oxidative stress HPV16E6 function might contribute to c-fos expression and c-fos/c-jun heterodimer formation. 

There are at least 15 cancer-associated HPV types, and of these, HPV16 is found in more than 50% of HPV-positive cancer tissues [1, 2]. Here, we used SiHa cells (HPV16-positive squamous cell carcinoma of the cervix) and showed that H_2_O_2_ and TGF-*α*, which are promoters of the multistep carcinogenic process [18, 19], induced c-fos/c-jun heterodimer formation in SiHa cells, whereas *o-*phenanthroline inhibited H_2_O_2_-induced c-fos upregulation. H_2_O_2_ and TGF-*α*-induced c-fos upregulation was impaired in SiHa cells transfected with siRNA HPV16E6. This result further implicates H_2_O_2_ and TGF-*α* signaling in the pathogenesis of HPV-induced tumor development.

## 2. Material and Methods

### 2.1. Reagents

H_2_O_2_ was purchased from WAKO (Tokyo, Japan), and *o*-phenanthroline was obtained from Sigma-Aldrich (S.t. Louis, Mo, USA).

### 2.2. Cell Culture and Treatment

The SiHa and Caski cell line used in our study was obtained from the American Type Culture Collection. SiHa cells were routinely cultured in DMEM (Invitrogen) supplemented with 5% fetal bovine serum (Sigma) at 37°C under 5% CO_2_. SiHa cells were serum-deprived for at least 16 hours before being stimulated with H_2_O_2_ (1 mM). To observe the effect of an Fe^2+^ chelating agent on the activation of c-fos, *o*-phenanthroline (0.2 mM) was added to the culture medium 1 hour before stimulation with H_2_O_2_.

### 2.3. SDS–PAGE and Western Blotting

The cell cultures (80–100% confluent) were washed twice with ice-cold phosphate buffered saline and lysed in 1 × Laemmli sample buffer supplemented with a protease inhibitor cocktail (Roche) and a phosphatase inhibitor cocktail (Roche). The cells were scraped and homogenized at 4°C with a syringe and a thin needle, and the cell lysates were subjected to sodium dodecyl sulfate-polyacrylamide gel electrophoresis (SDS-PAGE, 10% resolving gel). The resultant proteins were electrophoretically transferred onto nitrocellulose membranes (Pall Corporation), before being incubated for 2 h at room temperature with blocking buffer containing 4% nonfat dried milk in TBS (20 mM Tris (pH 7.6) and 150 mM NaCl). They were then washed with TBS-T (0.1% Tween-20) and incubated overnight at 4°C in TBS buffer containing 4% BSA (Wako) and appropriate antibodies. The antibodies used in the experiment were anti-c-fos (Santa Cruz, sc-52, 1 : 500) and anti-c-jun (Cell Signaling, L70B11, 1 : 500), and the secondary antibodies were Donkey Antirabbit IgG/HRP (Amersham NA934, 1 : 5000) and Goat Antimouse IgG/HRP (Chemicon, AP124P 1 : 5000).

The protein bands were visualized using an enhanced chemiluminescence (ECL) detection system (GE Healthcare Bio-Sciences). The signals from the membrane were detected and imaged using a LAS-4000 imager (Fujifilm Corporation). The membranes were then stripped (30 min at 56°C) in stripping buffer containing 100 mM 2-mercaptoethanol, 2% SDS, and 62.5 mM Tris–HCl (pH 6.7) and reprobed with *β*-actin or tubulin as a loading control. All Western blots were performed at least three times for each experiment. To quantify the Western blot data, densitometric analysis of ECL-exposed blots was performed using the NIH ImageJ software (version 1.45f).

### 2.4. CoIP Assay

The antibodies used for the Western blot analysis were c-Fos, c-Jun, and CBP/*β*. For the c-Fos coIP assay, SiHa cells were lysed in a modified RIPA buffer (50 mM Tris-HCl (pH 7.4), 150 mM NaCl, 1 mM EDTA, and 1% NP-40) and a protease inhibitor mixture (Roche). CoIP was performed using 2 *μ*g of the c-Fos antibody or the respective isotype control.

### 2.5. Preparation of Small Interfering RNA and Transfection

Synthesized siRNA duplexes were obtained from Invitrogen. The siRNA sequences targeting HPV16E6 corresponded to nucleotides 5′-ACCGUUGUGUGAUUUGUUATT-3′ (siRNA1-HPV16E6) and 5′-UAACAAAUCACACAACGGUTT-3′ (siRNA2-HPV16E6) of the coding region [20]. The sequence of the negative control (siRNA-scrambled) was as follows: 5′-CCAUUCCGAUCCUGAUCCGTT-3′ and 5′-CGGAUCAGGAUCGGAAUGGTT-3′. Cells in the exponential growth phase were plated in a 35 mm dish containing antibiotic-free medium at 30% confluence and then transfected with siRNA (siRNA-HPV16E6 or siRNA-scrambled) using Oligofectamine (Invitrogen) and Opti-MEM I (Invitrogen), according to the manufacturer's protocol. Silencing was examined 24 h after transfection.

### 2.6. RT-PCR

Total RNA were extracted from the harvested cells using Isogen (Nippon Gene). After the removal of genomic DNA, approximately 1 *μ*g of RNA was used to generate cDNA using Reverse Transcriptase M-MLV (RNase H-) (TaKaRa) and random hexamers (Fermentas). The primer sequences used were as follows: 5′-CGGAATTCATGCACCAAAAGCGAAC-3′ (sense) and 5′-CCCAAGCTTACAGCTGGGTTTCTCT-3′ (antisense) for HPV16E6 [21] and 5′-CAGGGCTGCTTTTAACTCTG-3′ (sense) and  5′-GATGATCTTGAGGCTGTTGTC-3′ (anti-sense) for GAPDH. The PCR reactions for HPV16E6 were run for 30 cycles. GAPDH amplification products were used as a loading control. All amplification products were separated in 2% agarose gels. All RT-PCR were performed at least three times for each experiment.

### 2.7. Statistical Analysis

Data are expressed as the mean ± s.e.m. The paired-sample *t*-test was performed for statistical comparisons. For all tests, a *P* value of less than 0.05 was considered to be significant.

## 3. Results

### 3.1. o-phenanthroline Inhibits H_2_O_2_-Induced c-Fos Expression

**Figure 1:**
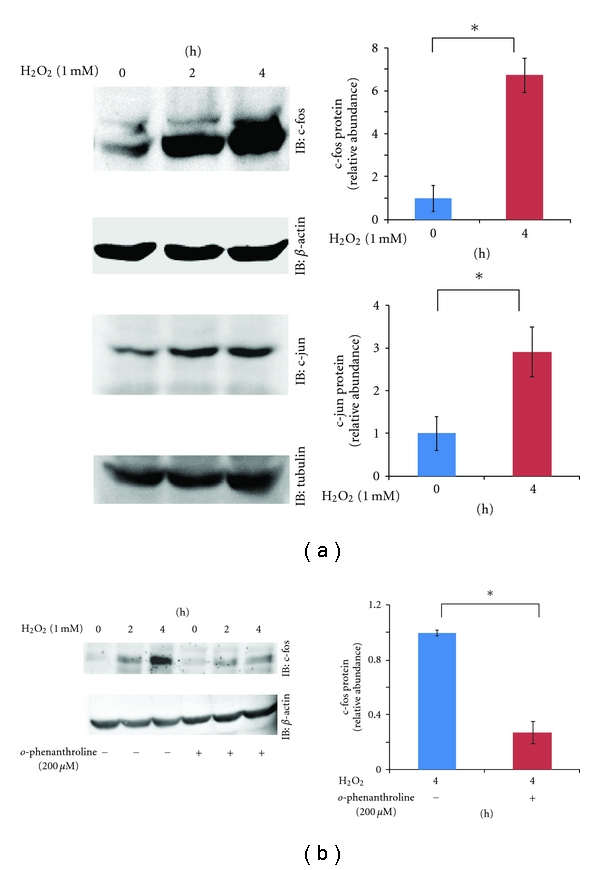
(a) Upregulation of AP-1 gene expression in SiHa cells at the indicated time points after H_2_O_2 _(1 mM) exposure. Representative blots are shown and include *β*-actin or tubulin as a loading control, along with the results of densitometric analysis (0 h, 4 h; normalized to *β*-actin or tubulin). *P < 0.05, *n* = 3. (b) Inhibitory effect of *o*-phenanthroline on H_2_O_2_-induced c-fos upregulation. Cells were pretreated for 1 hour with or without *o*-phenanthroline (200 *μ*M) before H_2_O_2_ (1 mM) exposure. Representative blots are shown and include *β*-actin as a loading control, along with the results of densitometric analysis (4 h; normalized to *β*-actin). *P < 0.05, *n* = 4.

The upregulation of c-fos has been observed after H_2_O_2_ treatment in both epithelial and endothelial cells [19, 22], which raises the possibility that SiHa cells might respond to H_2_O_2_ in a similar manner. To test this, we analyzed c-fos protein expression after H_2_O_2_ treatment in SiHa cells. Western blotting analysis revealed aberrantly high levels of c-fos protein expression after H_2_O_2 _treatment (Figure 1(a)). In the H_2_O_2_-treated SiHa cells, the expression of the AP-1 family member c-jun was also upregulated (Figure 1(a)). *o-*phenanthroline is an iron chelator [23] that protects cells from cancer progression by inhibiting the Fenton reaction, and it has been reported that in a mouse model of gastric cancer the intraperitoneal injection of *o-*phenanthroline significantly reduced the incidence of gastric cancers [24] by inhibiting the Fenton reaction. The effect of *o*-phenanthroline on c-fos expression in SiHa cells is unclear; therefore, we tested the effect of *o-*phenanthroline on H_2_O_2_-induced c-fos expression in SiHa cells by pre-treating SiHa cells with *o-*phenanthroline before H_2_O_2 _treatment. As a result, we found that *o-*phenanthroline markedly inhibited H_2_O_2_-induced c-fos expression (Figure 1(b)).

### 3.2. Characterization of c-Fos/c-jun Heterodimers after Ligand-Dependent and Independent EGFR Activation

**Figure 2:**
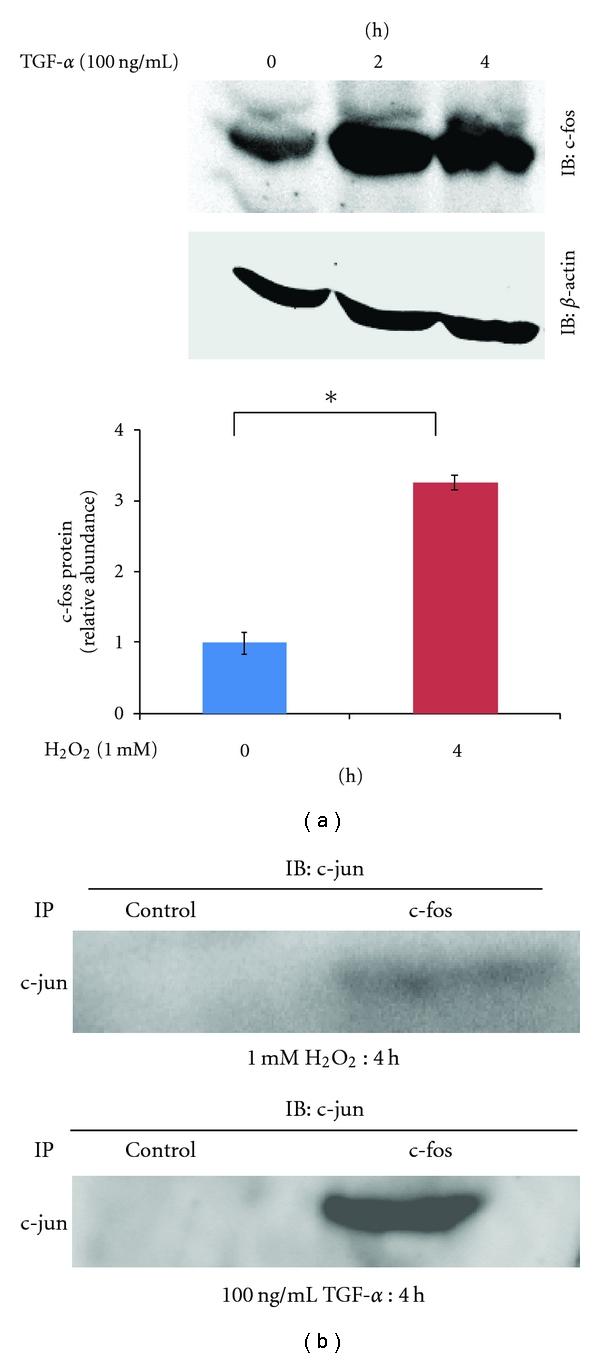
(a) c-fos upregulation in SiHa cells after TGF-*α* (100 ng/mL) treatment. Cells were harvested at the indicated time points and used for Western blotting analysis. Representative blots are shown and include *β*-actin as a loading control, along with the results of densitometric analysis (0 h, 4 h; normalized to *β*-actin). *P < 0.01, *n* = 3. (b) IP of c-fos was performed using whole-cell extracts. Coprecipitated proteins were detected by Western blotting analysis using antibodies specific to c-jun.

The upregulation of c-fos observed in epithelial cells after H_2_O_2_ treatment is mainly mediated through EGFR activation [18]. We, therefore, examined c-fos protein expression in SiHa cells that had been treated with the EGFR ligand TGF-*α*. Western blotting analysis revealed aberrantly high levels of c-fos protein expression after TGF-*α* treatment (Figure 2(a)). We then investigated c-fos/c-jun heterodimer formation in SiHa cells. We stimulated SiHa cells with H_2_O_2_ or TGF-*α* for 4 hours, immunoprecipitated c-fos, and determined its partner AP-1 family member by Western blotting (Figure 2(b)). Interactions between c-fos and c-jun were observed after both H_2_O_2_ and TGF-*α* stimulation (Figure 2(b): the IP with isotype control did not show any specific band).

### 3.3. HPV16E6 Mediates H_2_O_2_ and TGF-*α*-Induced c-Fos Expression

**Figure 3:**
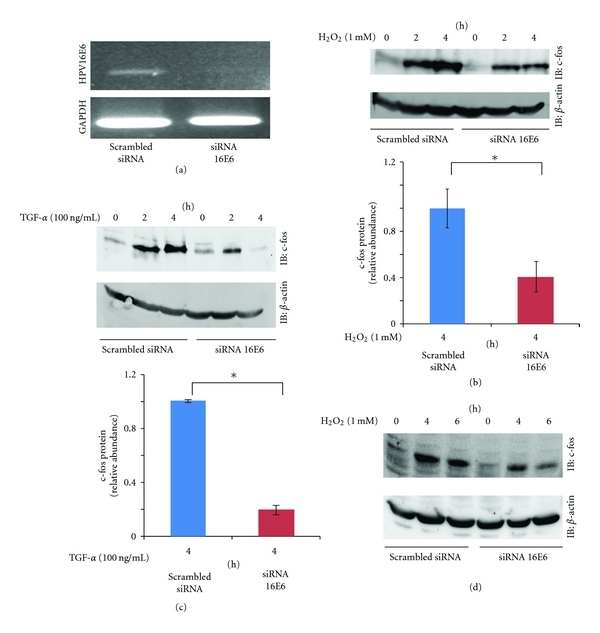
HPV16E6-mediated upregulation of c-fos in SiHa cells. (a) The mRNA levels of HPV16E6 were detected by RT-PCR after HPV16E6 siRNA transfection into SiHa cells. Cells were transfected with scrambled siRNA or HPV16E6 siRNA for 24 hours. (b) HPV16E6-mediated upregulation of c-fos expression in SiHa cells after H_2_O_2_ exposure. The transfected cells were exposed to H_2_O_2_ (1 mM) and harvested at the indicated time points, before being subjected to Western blotting analysis. Representative blots are shown and include *β*-actin as a loading control, along with the results of densitometric analysis (4 h; normalized to *β*-actin). *P < 0.05, *n* = 4. (c) HPV16E6-mediated upregulation of c-fos expression in SiHa cells after TGF-*α* exposure. Transfected cells were exposed to TGF-*α* (100 ng/mL) and harvested at the indicated time points, before being subjected to Western blotting analysis. Representative blots are shown and include *β*-actin as a loading control, along with the results of densitometric analysis (4 h; normalized to *β*-actin). *P < 0.05, *n* = 4. (d) HPV16E6-mediated upregulation of c-fos expression in Caski cells after H_2_O_2_ exposure. The transfected cells were exposed to H_2_O_2_ (1 mM) and harvested at the indicated time points, before being subjected to Western blotting analysis.

We next tested whether HPV16E6 is necessary for the c-fos expression induced by H_2_O_2_ or TGF-*α* treatment. To directly address the role of HPV16E6 protein in c-fos expression, we transfected SiHa cells with siRNA HPV16E6 (Figure 3(a)). A scrambled nonspecific siRNA duplex was used as a negative control. We observed siRNA HPV16E6-dependent reductions in H_2_O_2_ and TGF-*α* induced c-fos expression (Figures 3(b) and 3(c)). Similar results that siRNA HPV16E6-dependent reduction in c-fos expression induced by H_2_O_2_ treatment were obtained in another cervical cancer cell line, Caski cell (Figure 3(d)). These data suggest that HPV16E6 is necessary for H_2_O_2_ and TGF-*α* induced-c-fos expression.

## 4. Discussion

Our findings provide new insights into the mechanism of c-fos/c-jun heterodimer formation during the development of cervical cancer. First, our findings show that c-fos/c-jun heterodimer formation results from both H_2_O_2_ and TGF-*α* stimulation. The current view is that in cervical cancer c-fos/c-jun heterodimer formation results from the inefficient expression of the ternary complex factor Net [25], but the evidence we have uncovered for HPV16E6 dependent c-fos expression after H_2_O_2_ or TGF-*α* stimulation broadens the scope of the mechanisms of c-fos/c-jun heterodimer formation in extracellular conditions such as oxidative stress. For example, the observed shift in AP-1 composition from fra-1/c-jun to c-fos/c-jun heterodimers in HaCaT keratinocytes after UV-B exposure [10] suggests that after being induced by extracellular and environmental stimuli, c-fos expression and c-fos/c-jun heterodimer formation contribute to the pathological process of HPV positive skin cancer. Therefore, studies of c-fos expression, HPV infection, and skin tumor development are necessary.

Second, our data indicate that HPV16E6 is necessary and sufficient for c-fos/c-jun heterodimer formation to occur in SiHa cells under both H_2_O_2_ and TGF-*α* stimulation. In the presence of transition metals such as iron and copper, H_2_O_2_ can be oxidized into extremely reactive and toxic HO• via the Fenton reaction. The hydroxyl radical (HO•) then activates EGFR [26]. Our data show that H_2_O_2_ and TGF-*α* are stimulators of c-fos/c-jun heterodimer formation and confirm that the iron chelator is an inhibitor of c-fos expression that acts by inhibiting the Fenton reaction. The negative effect of *o*-phenanthroline on HPV16E6 mediated c-fos expression might explain why iron chelation inhibits growth and induces the apoptosis of HPV-positive carcinoma cells [27]. Tumorigenic cervical cells express higher levels of intracellular iron [28], which creates an environment that favors HPV16E6-mediated c-fos/c-jun heterodimer formation in HPV-infected cells. This implies that iron is necessary for c-fos/c-jun heterodimer formation. The precise triggers of H_2_O_2_ expression around cervical epithelial cells are unknown, but it has been proposed that chronic inflammation triggers the accumulation of macrophages in the vicinity of the cervix, and that these cells subsequently produce the antimicrobial molecule H_2_O_2_. 

Finally, our data indicate that cervical cancer, the hallmark of which is HPV infection, is an EGFR-sensitive disease regardless of ligand-dependent or ligand-independent stimulation. EGFR mAb therapies are effective for treating squamous cell carcinoma of the head and neck and are also being evaluated in other HPV-associated tumors [29]. One of the best-known mechanisms of EGFR mAb therapy resistance is ligand-independent EGFR activation [30]. This was observed in studies of squamous carcinoma cells (T-Hep3) that naturally overexpress the uPA receptor, one of which found that EGFR was activated by the uPA receptor in a ligand-independent fashion, resulting in resistance to EGFR mAb [31]. Therapies such as the use of *o-*phenanthroline, or alternative approaches such as the use of small molecule EGFR tyrosine kinase inhibitors that block the intracellular tyrosine kinase domain, could hold great potential for the treatment of head and neck cancer.
